# The influence of pitch feedback on learning of motor -timing and sequencing: A piano study with novices

**DOI:** 10.1371/journal.pone.0207462

**Published:** 2018-11-28

**Authors:** Claudia Lappe, Markus Lappe, Peter E. Keller

**Affiliations:** 1 Department of Medicine, Institute for Biomagnetism and Biosignalanalysis, University of Muenster, Münster, Germany; 2 Institute for Psychology & Otto Creutzfeldt Center for Cognitive and Behavioral Neuroscience, University of Muenster, Münster, Germany; 3 The MARCS Institute for Brain, Behavior and Development, Western Sydney University, Penrith, Australia; University College London, UNITED KINGDOM

## Abstract

Audio-motor coordination is a fundamental requirement in the learning and execution of sequential actions such as music performance. Predictive motor control mechanisms determine the sequential content and timing of upcoming tones and thereby facilitate accurate performance. To study the role of auditory-motor predictions at early stages of acquiring piano performance skills, we conducted an experiment in which non-musicians learned to play a musical sequence on the piano in synchrony with a metronome. Three experimental conditions compared errors and timing. The first consisted of normal auditory feedback using conventional piano key-to-tone mappings. The second employed fixed-pitch auditory feedback consisting of a single tone that was given with each key stroke. In the third condition, for each key stroke a tone was randomly drawn from the set of tones associated with the normal sequence. The results showed that when auditory feedback tones were randomly assigned, participants produced more sequencing errors (i.e., a higher percentage of incorrect key strokes) compared to when auditory feedback was normal or consisted of a single tone of fixed pitch. Furthermore, synchronization with the metronome was most accurate in the fixed-pitch single-tone condition. These findings suggest that predictive motor control mechanisms support sequencing and timing, and that these sensorimotor processes are dissociable even at early stages of acquiring complex motor skills such as music performance.

## Introduction

Sensorimotor synchronization is a critical aspect of playing a musical instrument. Temporal and spatial precision of actions, for example the finger and hand movements of a pianist, are necessary to achieve an accurate and satisfying performance [1,2]. When playing an instrument, such movements are guided by expectations and predictions for upcoming tones and also generate predictions about subsequent acoustic events. Two kinds of predictive mechanisms are relevant in music performance: first, motor predictions in which a finger movement predicts the following tone, and second, melodic predictions, in which auditory expectations arise from the musical structure of a melodic sequence [3,4].

Anticipating the auditory consequences of finger movements enables musicians to adapt and correct their movements during performance so that errors can be avoided [5]). A comparison of these predictions with the actual auditory outcome represents a mechanism to acquire a fluent musical performance over the course of training [6,7]. In addition, auditory feedback guides error correction and helps to build the sensorimotor mapping between movements and their sensory consequences [8]. This establishes internal forward models that link efference copies of specific motor acts to associated musical tones to produce associations between motor actions and their auditory consequences [9,10,11]. In such a forward model, the motor command to press a specific key on the instrument is used by the brain to predict the auditory effect (pitch and time) of pressing that key. The comparison of this prediction to the actual auditory input after the key stroke produces an error signal. The error signal is used to adjust the motor command before the movement is executed. In this way, predictive processes are used to monitor performance based on congruency of auditory feedback with forward model predictions.

Links between predictive processes and the accuracy and fluency of musical performance have been investigated using altered feedback paradigms. In such experiments, timing or pitch content of auditory feedback from piano key strokes is typically manipulated [12–16]. For example, experiments testing delayed auditory feedback (i.e., a constant time lag between the key stroke and its auditory outcome) showed significant disruptive effects on timing in musical performance [13,17]. Longer delays, for example when auditory feedback was given only after the next key was pressed (i.e. a one-back feedback) also produced errors in sequencing [14,15]).

Alterations of feedback pitch content, on the other hand, appeared to have no disruptive effects for timing or sequencing [18]. Performance of well-rehearsed musical sequences was largely unimpaired when auditory feedback was removed [19], when auditory feedback corresponded to a transposed version of the sequence [20], or even when pitch feedback was random and unpredictable [18,21]. Such findings imply that in well rehearsed and memorized musical sequences the presence of auditory feedback might not be critical [19], perhaps because such sequences are directly supported by pure motor memory or vivid and robust auditory imagery (Keller, 2012) [22]. However, auditory feedback is likely important for the acquisition of musical performance abilities [5,6].

The purpose of the present study was to investigate the role of prediction in the newly acquired skill of playing a musical melody in synchrony with a metronome (which imposes strict constraints on performance tempo). We hypothesized that a match in pitch between a planned finger movement with the auditory outcome assists in sequence learning and timing of performance. For this purpose we tested non-musicians in three conditions. The first condition presented normal pitch feedback such that associations between keys and tones conformed to the culturally familiar mapping of low-to-high pitches from left-to-right on a keyboard [23,24,25] and the participants’ knowledge of melodic progression in western musical style that is acquired through mere exposure [26,27]. The second condition presented the same fixed pitch for each key stroke such that auditory feedback was highly predictable, which was expected to be useful for timing but not sequencing. The key strokes in this condition were, however, not related to the particular key that was pressed nor to the melodic sequence. In the third condition, at each key stroke a pitch was randomly drawn from the set of pitches in the sequence so that auditory feedback content was unpredictable.

We analyzed performance in each condition with respect to sequencing errors and temporal parameters. The percentage of correctly performed sequences was used as the measure for sequencing accuracy, and smoothness of playing tempo was assessed by evaluating inter onset intervals (IOIs) and their variability. Synchronization with the metronome, a further requirement of the task, was quantified by analyzing the temporal offset of each key stroke from the nearest metronome beat. It was assumed that synchronization would be high to the extent that participants prioritize timing (temporal prediction) over sequencing (melodic prediction), as may be the case when auditory feedback is uninformative.

## Methods

### Participants

Twenty-nine (18 female) non-musician students, aged between 17 and 38 years, from Western Sydney University, participated in the study in exchange for course credit. One participant reported being left-handed, all other participants were right handed and none had a history of ontologically or neurologically related disorders. Participants had little or no formal musical training. None of the participants had received piano lessons.

No participant displayed impairment in motor skills and all were able to perform the task motorically. Participants were fully informed about the nature of the study and gave written informed consent to take part in it. For participants under the age of 18 years, informed consent was obtained from the participant and from their parents. The experimental procedures conformed with the Declaration of Helsinki and were approved by the Human Research Ethics Committee at Western Sydney University.

### Materials

**Fig 1:**
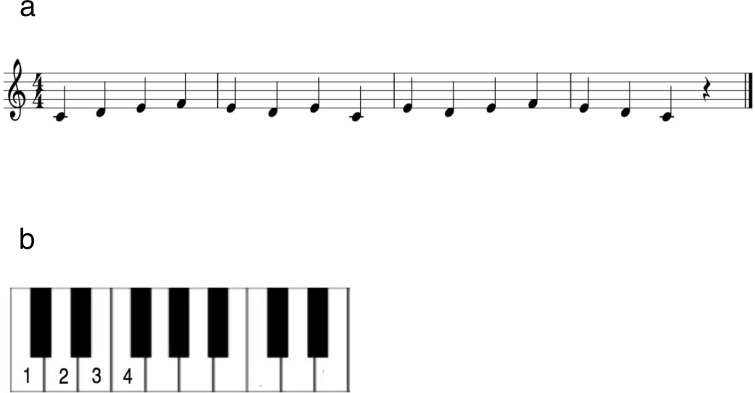
a: The musical sequence played by the non-musician participants. b: Visual template on which the image of the piano keyboard was depicted and the finger placements were marked. Numbers represent the fingers (thumb, 1; index finger, 2; etc) with which the participants were supposed to press the corresponding piano keys.

A melody comprising 15 notes was used as stimulus material (Fig 1). The melodic sequence was notated in a binary meter and in the key of C-major. The isochronous melodic line ranged from C to G containing no repeating pitches. The melody consisted mostly of whole and half steps (i.e., tones and semitones) with only two intervals spanning a major third (four semitones) in the middle of the sequence (Fig 1A). To facilitate the task a visual template was provided on which the piano keys and finger placements were depicted (Fig 1B).

The experiment was conducted on a weighted-key digital Yamaha Clavinova, to which an iMac laptop was connected via USB cable. Auditory feedback was presented through the piano’s speakers but manipulated via MIDI connection through a custom made program written in Objective C. The program also recorded and stored the key strokes on the computer. In the melodic feedback condition auditory feedback was normal and the tones sounded as they were played. In the single tone (‘fixed-pitch’) feedback condition, auditory feedback was a single tone (the note a’) following each key stroke. In the random feedback condition auditory feedback consisted of a tone randomly drawn from the set of tones of the correct sequence (C,D,E,F). This was done independently at each key stroke resulting in a new random sequence of tones in each trial. Metronome beats at a steady rate of 2Hz consisted of short click sounds that were generated and controlled by the computer program.

### Procedure

An experimenter instructed the participant how to play the sequence. Participants practiced the melodic sequence for about 10 minutes with normal auditory feedback in order to memorize it and performed smoothly. Participants then further practiced for another five minutes to play the melodic sequence in synchrony with the metronome at a rate of 2Hz. Thereafter data collection started. Auditory feedback conditions were performed in blocks and counterbalanced between participants. In each block, 20 sequences were played successively. At the beginning of each block the metronome was started and participants began playing along the beat whenever they felt ready. Participants were instructed to separate the sequences by allowing one intermediate metronome beat between two consecutive sequences. Between blocks participants were allowed to take a short break for stretching or adjusting their seating posture. Each block was repeated 4 times resulting in 80 sequences for each feedback condition and 240 sequences altogether. The entire experimental session lasted about 40 min. Piano performance was recorded via MIDI and stored on the computer. Twenty-five out of the total of 348 blocks (7%) were affected by technical issues with the data collection and could not be analyzed, leaving 6460 sequences for analysis.

### Data analysis

From the experimental recordings five performance parameters were analysed: the percentage of correctly played sequences, the mean and standard deviation of the inter onset intervals (IOIs) between two successive key strokes, and the mean and standard deviation of the temporal offset to the metronome. Data analysis was performed using the software package Mathematica 9. Statistical analysis were done in R. Performance errors were detected with a software program in which the recorded MIDI note numbers of key strokes of the performance were compared with the MIDI numbers associated with the correct sequence.

Accuracy of timing was examined by calculating the mean of the produced IOIs and the mean of the absolute phase difference with regard to the metronome. Two measures of synchronization accuracy with the metronome were computed: the mean signed asynchrony (which is informative about whether key strokes led or lagged behind metronome clicks, on average) and the mean unsigned asynchrony (a more general measure of accuracy). Precision of timing was measured via the standard deviation of the IOI and phase.

The five dependent measures were entered into separate one-way repeated-measures analyses of variance (ANOVAs) to test for effects of feedback condition (normal, single-tone, random).

## Results

**Fig 2:**
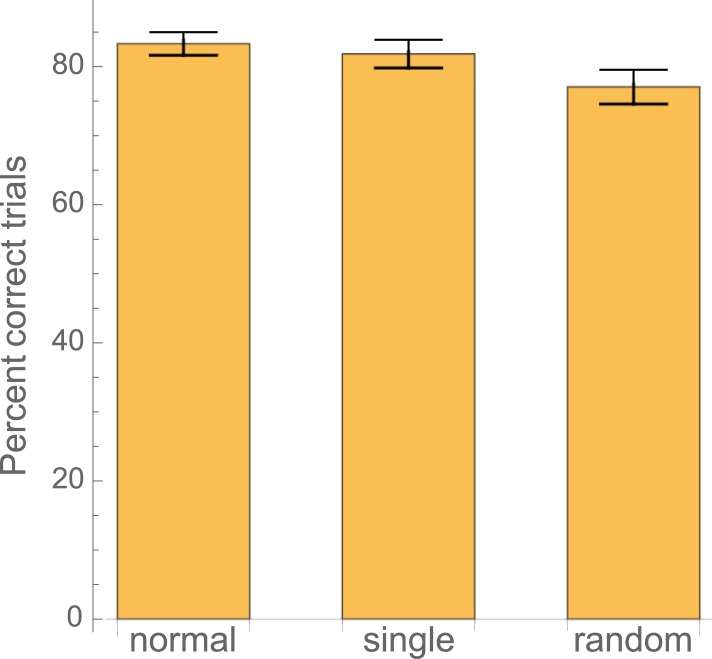
The percentage of correctly played sequences: Subjects made significantly more mistakes when feedback was not predictable.

Fig 2 shows the percentage of correctly performed sequences in each of the three conditions. The ANOVA on these data revealed a statistically significant main effect of condition (F(2,56) = 5.36; p = 0.0073) and post-hoc pairwise comparisons showed that accuracy in the random condition was significantly lower than in the normal condition (p = 0.0071) and marginally lower than in the single tone condition (p = 0.063). No difference in accuracy was found between the normal and the single tone condition.

**Fig 3:**
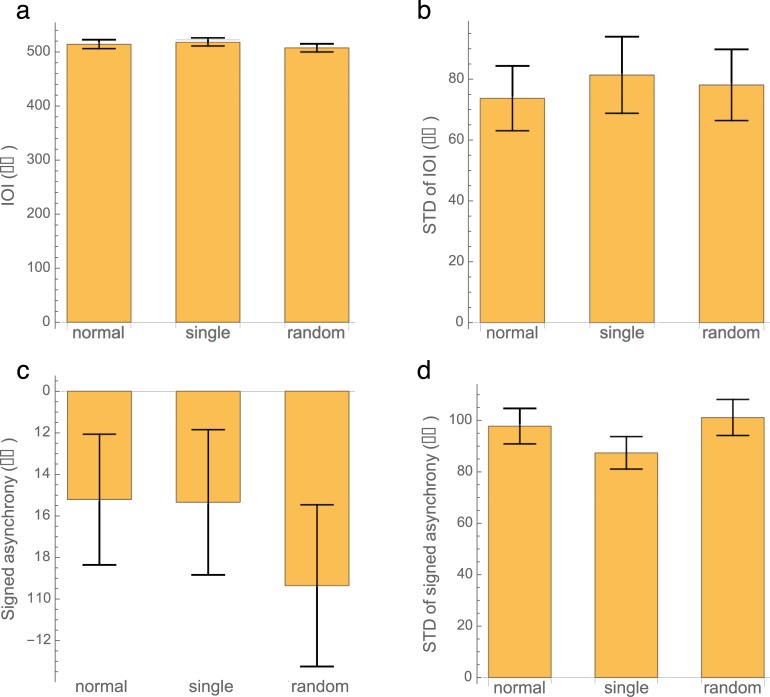
a/b: Inter onset interval (IOI) and the standard deviation (STD) of the inter onset interval. No significant difference was found between conditions. 3c: The signed asynchrony to the nearest metronome beat. There was no difference between conditions. Participants’ key strokes slightly preceded the metronome beats. 3d: The standard deviation of the signed asynchrony. Variability in the single tone condition was lower than in the random and normal conditions.

The ANOVA on IOIs (Fig 3A) yielded no significant difference between conditions (F(2,56) = 2.0; p = 0.14), nor did the ANOVA on the standard deviation of the IOIs (Fig 3B; F(2,56) = 0.58; p = 0.56).

Fig 3C shows the signed asynchrony to the nearest metronome beat for the three conditions. On average, participants’ key strokes slightly preceded the metronome beats. The ANOVA did not show any significant difference between conditions (F(2,56) = 1.24; p = 0.30). Fig 3D shows the standard deviation of the signed asynchrony. The ANOVA on these data revealed a significant main effect of condition (F(2,56) = 9.61; p = 0.0002). Post-hoc pairwise comparisons revealed that variability in the single tone condition was lower than in the random condition (p = 0.0011) and lower than in the normal condition (p = 0.006).

## Discussion

We investigated how predictability of auditory feedback facilitates sensorimotor piano playing in novices. We compared a normal melody condition, in which the upcoming tones were predictable and could be deduced from conventional musical regularities, to a single tone condition with fixed pitch, in which the content of the upcoming tone was predictable but uninformative about which key had been pressed, and a random condition, in which the pitch of the auditory feedback was randomly drawn from the set of tones in the sequence and therefore no reliable prediction regarding pitch content was possible. The results revealed a higher rate of sequencing errors in the condition in which auditory feedback was randomly assigned compared to when feedback was normal. Secondly, while speed and smoothness of performance timing were not affected by feedback condition, synchrony with the metronome was most accurate in the single tone condition.

Randomly assigned pitch feedback presumably increased sequencing error rates because variable feedback does not present the performer with a consistent match between a key stroke and a specific piano tone or with a coherent melodic structure. This may have been detrimental to performance to the extent that, under such conditions, expectations for upcoming tones cannot be formed and a reliable internal forward model cannot be established [10]. In the normal feedback condition, on the other hand, pure motor prediction for the following tone as well as musical prediction for the melodic progression may have been used to improve accurate sequencing during performance. The observed difference between the two conditions thus supports a role of pitch feedback in movement sequencing.

While the single tone condition supports motor prediction, since the act of pressing a key produces a predictable sound, the pitch of that sound does not provide feedback about the particular key that was pressed nor about the progression of the musical sequence. Sequencing results in that condition did not differ from the normal condition, although a trend towards poorer performance was observed. These results suggest that the availability of motor prediction may help to produce proper sequencing and that musical prediction may be less involved in sequencing.

The results of single tone condition are also inconsistent with models that use auditory context from preceding tones in the learned sequence as a predictor of upcoming tones. In the normal sequence each tone provides context for the next tone and can be used to predict the next pitch. This leads to better performance in the normal than in the random condition. However, in the single tone condition no such contextual associations can be formed since the sequence of fixed pitches is uninformative with regard to the keys that have to be pressed. Hence, this model would predict that performance in the invariant condition should be worse than in the normal condition. This was not the case.

Our finding that sequencing was disturbed by random auditory feedback differs from previous studies that found no such effect and, accordingly, argued that performance fluency does not necessarily require online action-perception coupling [6,15,18,19,21]. However, these studies were typically conducted with musicians playing well-rehearsed test material. In that case, the participants’ motor programs may have allowed the movements to be executed automatically, such that auditory feedback was not necessary anymore. Alternatively, anticipatory musical imagery, which is more advanced in musicians [7], might have guided the performance and substituted the actual correct auditory feedback [28]. Pfordresher (2005) [21] compared musicians and non-musicians using normal and random feedback and reported no differences between conditions and groups. However, the sequence in that study was shorter than ours (8 instead of 15 tones), possibly making the task easier and therefore leading to comparable accuracy levels in musicians and non-musicians.

Herrojo Ruiz et al. (2017) [16] investigated effects of altered auditory feedback in piano novices and found that random feedback did not produce higher error rates, similar to Pfordresher et al. (2014) [15] in trained musicians. However, the random auditory feedback conditions in those studies were different from that of our study, as they presented pitches outside the range of the normal sequence while we presented pitches selected randomly from within the normal sequence. Consistent with our results, Herrojo Ruiz et al. (2017) [16], Pfordresher et al. (2014) [15], and Pfordresher (2005) [21] found increased error rates when they presented pitches from within the normal sequence in altered serial order. In addition to interfering with sensorimotor prediction, such serially shifted feedback may also produce interference by inducing the formation of new and competing representations of the sequence if the new serial order is consistent across trials [21]. However, our random condition assigned a new pitch at each keypress. Thus, the sequence was different in every trial and unlikely to support the establishment of a stable competing representation. Therefore, our results support the notion that motor prediction helps to produce proper sequencing in sequential actions [15].

The second set of findings of the present study relates to disruption of timing: while performance speed (the average time interval between key strokes) and smoothness (the variability of inter-key stroke intervals) were unaffected by feedback condition, phase coherence (mean asynchrony) and phase precision (STD of asynchronies) displayed a consistent pattern of variation between conditions. These findings are remarkable because temporal aspects of feedback were identical in all conditions. The results hence suggest that the accuracy and precision of timing relative to a metronome are affected by pitch feedback.

Synchronization with the metronome was more precise in the single tone compared to the melody and random condition. In the single tone condition, prediction involved only timing of feedback since pitch content was always the same. The prioritization of timing in this condition was presumably conducive to meeting the timing demands of synchronizing with the metronome. In the normal melody condition, however, prediction involves timing and pitch content. The process of comparing sensory outcomes to predictions might operate on both aspects and the comparison of pitch may interfere with timing and therefore hamper timing accuracy [29]. Such interference would also explain why phase coherence was low in the random condition. An automatic and mandatory processing of pitch feedback may interfere with timing in this condition since the pitch feedback cannot be predicted at all.

Taken together, these findings indicate that timing was most accurate in the single tone condition in which predictions between the hand movement and a piano tone were possible but prediction based on melodic progressions was impossible [10,30,31]. Therefore, as is the case with sequencing, accurate timing depends on motor prediction.

The results demonstrate that sequencing and timing are not completely independent. Previous studies investigating sequencing and tempo effects of auditory feedback alterations in music performance had reported a dissociation: alteration of pitch content disrupted sequencing but left timing unimpaired [21], whereas temporal asynchronies between key strokes and auditory feedback disrupted timing but not sequencing [14]. Our results on sequencing and tempo are consistent with these findings, but our measurements of phase coherence with a metronome showed that pitch feedback can also influence temporal parameters.

## Conclusion

The results in the present study revealed that non-musicians produce more sequencing errors on a newly learnt piano playing task when auditory feedback is random and unpredictable than with normal feedback. Performance timing (i.e., synchrony with the metronome), on the other hand, was most accurate in a stereotyped condition, in which only single one tone was given as auditory feedback. These findings suggest that, at least in musical novices, motor predictions relating to upcoming key strokes support proper sequencing and timing, while melody based predictions are less involved. Our results furthermore showed interference effects between sequencing and timing, suggesting that these sensorimotor processes are not completely independent even in the case of a newly acquired motor skill.
